# Interface Controlled Electric Field Swing Adsorption

**DOI:** 10.1002/advs.202504617

**Published:** 2025-07-03

**Authors:** Silvio Heinschke, Jörg J. Schneider

**Affiliations:** ^1^ Technische Universität Darmstadt Eduard‐Zintl‐Institut für Anorganische und Physikalische Chemie Peter‐Grünberg‐Str. 12 64287 Darmstadts Germany

**Keywords:** activated carbon, activated carbon, electrified gas adsorption, field gradient, gas desorption

## Abstract

Influencing the adsorptive processes of gases by external stimuli is an ongoing research task of academic and technological relevance. Technologically external stimuli like pressure, vacuum, temperature, magnetic field, or electrical phenomena are the most common ones with which adsorptive and desorptive processes can be influenced. In the case of pure electric field swing adsorption (EFSA) of solid/gas mixtures, however, experimental knowledge concerning carbon materials is lacking so far. A new approach to the electrical field effect on gas adsorption and desorption is presented. Ar, N_2_ and CO_2_ interact with an all‐solid composite material composed of activated porous carbon and silica characterized by a high amount of charged interfaces under isothermal conditions and ambient temperature. The intimate contact of both components in the composite allows for the formation of multiple resistor‐conductor interfaces enabling the reversible physisorption of these gases using electric fields in the lower *V* and *mA* range. The adsorptive/desorptive swing effect depends on the polarizabilities of the gases in particular their dipoles and to an even larger extent on the field induced quadrupole moments of the probe gases Ar, N_2_ and CO_2_.

## Graphical abstract

Swing adsorption of Ar, N_2,_ and CO_2_ under moderate electric fields and elevated pressures at room temperature is investigated. Multiple charged conductor‐insulator interfaces enable a selective adsorption behavior of the three gases while CO_2_ shows a 10 fold increase in the effect over Ar and N_2_.

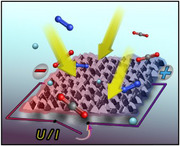

## 1 Introduction

**Figure 1:**
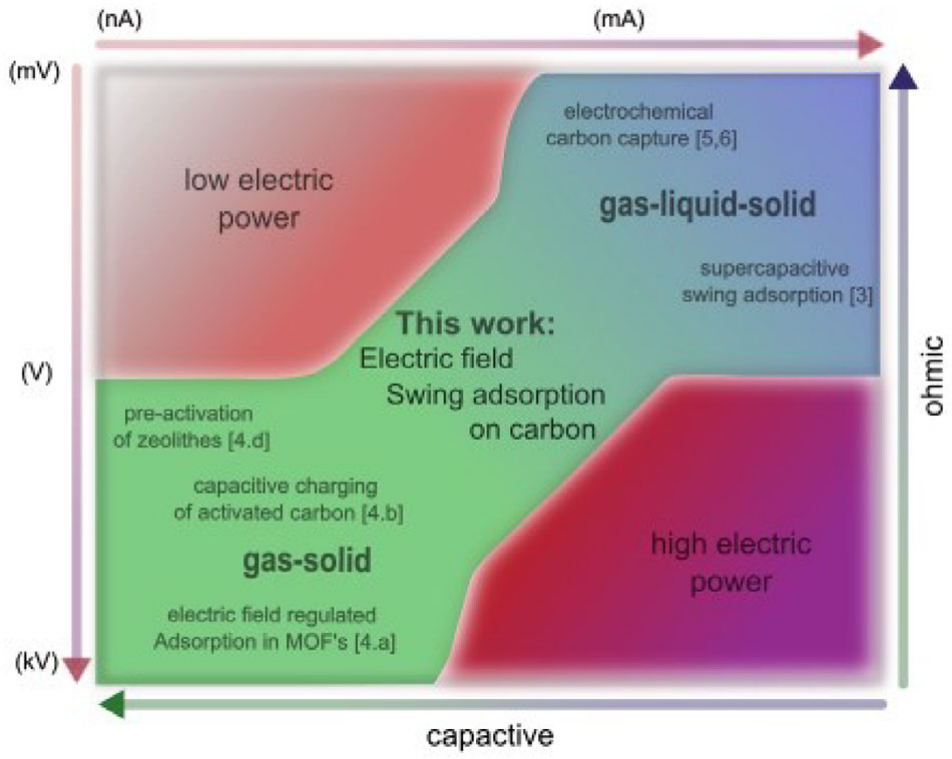
Categorization and comparison of current approaches to electrified gas adsorption in ECSA and EFSA processes.

Adsorption processes of gases in solid materials are crucial for almost any type of heterogeneous gas‐solid interaction. The adsorption of a gas or a gas mixture relies on the interface energy of the gas‐solid contact and the active outer and inner surface (e.g., pore type) of the solid sorbent. While the latter is pre‐determined by the individual material chosen and cannot be changed during the adsorption‐desorption process, the formers adsorption and desorption behavior may be influenced during both processes. A recent approach to achieve this goal is the electrification of the adsorptive process. Herein, three major directions have evolved:^[^ ^1^ ^]^ Electric Swing Adsorption^[^ ^2^, ^3^ ^]^ (ESA), Electrochemical Swing Adsorption including Supercapacitive Swing Adsorption (ECSA),^[^ ^4^ ^]^ and EFSA.^[^ ^1^ ^]^ ESA uses the increased desorption of gases due to heat evolvement of a flowing current, and ECSA the formation of molecular bonds by electrochemical interactions. In ESA, typically cascades involving resistor‐conductor‐mixtures have been investigated with respect to an application in cyclic separation processes.^[^ ^2^, ^3^ ^]^ Based on its versatility, stability and availability, activated carbon is one of the most investigated materials in the field of gas adsorption.^[^ ^5^, ^6^, ^7^ ^]^ Recent experimental and theoretical research with activated carbon is aiming to understand the structure and pore appearance in order to influence and foster the ability to capture greenhouse gases like CO_2_.^[^ ^8^, ^9^, ^10^, ^11^ ^]^ So far studying the behavior of carbon materials under the influence of electrical fields in gas/solid‐systems, is hampered by their comparably high conductivity when compared to pure non conducting oxide adsorbents like zeolites or coordination polymers like MOFs (metal organic frameworks) which have attained the largest interest in recent years.^[^ ^12^, ^13^, ^14^, ^15^, ^16^ ^]^ This might be because carbon materials, although probably the most widely used technologically relevant adsorbents for gases, tend to discharge severly even at low field gradients thus rendering carbon complicated to deal with causing side reactions followed by a deterioration of the carbon material.^[^ ^13^ ^]^ In the realm of ECSA, thus studies of gas‐liquid‐solid systems using activated carbon electrodes^[^ ^4^, ^17^, ^18^ ^]^ or reactive adsorption processes are scarce, quinone‐mediated CO_2_ capture^[^ ^19^, ^20^ ^]^ is one recent example. In EFSA, adsorption and desorption are regulated by applying an external electric field to a substrate to modify its absorptivity. Thus, a regulated interaction and polarization of adsorbent and adsorbate is created in which purely capacitive approaches (i.e., very low currents) and typically dielectric materials are dominant.^[^ ^12^, ^13^, ^14^, ^15^, ^16^ ^]^ Thus, EFSA differs from all other processes mentioned above in that only the field gradient induces adsorptivity/desorptivity effects. Interestingly, for EFSA processes besides purely theoretical work on 2D layered materials like BN, penta‐C_2_N (penta‐graphene containing C and N atoms),^[^ ^21^, ^22^ ^]^ high surface carbon materials like ordered mesoporous carbon^[^ ^1^ ^]^ or granular bituminous coal‐based amorphous carbon^[^ ^1^, ^23^ ^]^ experimental work concerning carbon materials seems completely lacking so far. Thus, the fundamental experimental effect of electrical charge on the adsorption behaviour of carbon is unknown. The usage of varying electrical fields in adsorption processes offers the future advantage that the adsorption/desorption process can be dynamically controlled by external means. **Figure** **1** shows an overview of the current state of the art.

To study the EFSA‐effect on carbon at elevated pressures experimentally for the first time we applied a 1:1 mixture by weight of the amorphous carbon Norit RX 1.5 Extra (in the following Norit) and SiO_2_ (see Experimental). Norit contains micropores which allow for an internal surface area of ≈1100 m^2^ g^−1^. Its external surface however is only 300 m^2^ g^−1^. The addition of SiO_2_ to the activated carbon host is used to modulate the conductivity of Norit to a level where the heat evolution of the flowing current can be fully compensated by externally applied cooling. This keeps the sample under an isothermal state. In that way Joule heating is suppressed, and a constant temperature is maintained which allows to study the influence of the external electric field on the adsorptive behaviour of the Norit. This allows to investigate the ability of an isothermal gas‐solid adsorptive process in which the electrical behaviour of the active carbon adsorbent is moderated by the dielectric material SiO_2_ leading to a characteristic excess adsorption of the gases used. Ball milling was used to produce the intimate mixture of both components generating many conductor‐resistor‐interfaces in the Norit/SiO_2_ composite. Application of a moderate electrical potential in the lower voltage range generates a current in the range of only a few mA resulting in an applied electric power of ≈0.55 W. The applied electrical power thus acts as a constant source of interface polarization of the sample.

## 2 Results and Discussion

### 2.1 Experimental Validation and Microscopic Model of the EFSA‐Effect

The activated carbon Norit used in this study is a strongly disordered material, however with a well‐characterized porous structure, with typically complex pore architecture and partially graphitic like character^[^ ^6^, ^25^, ^26^ ^]^ Several theoretical approaches to describe the structure are known.^[^ ^27^, ^28^ ^]^ Investigations of adsorption processes are focussed on the influence of pore size and structure, chemical modification and adsorbent temperature, pressure or novel parameters like rotation speed.^[^ ^1^, ^7^, ^29^, ^30^, ^31^, ^32^, ^33^, ^34^ ^]^ Such an activated carbon owes a type I isotherm^[^ ^35^, ^36^ ^]^ (see Section S1.7.2, Supporting Information) and is characterized as microporous material with a specific surface of ≈1470 m^2^ g^−1^. The SiO_2_ used shows a type IV isotherm^[^ ^35^, ^36^ ^]^ indicating a minor amount of micropores and a low specific surface area ≈ 340 m^2^ g^−1^.

**Figure 2:**
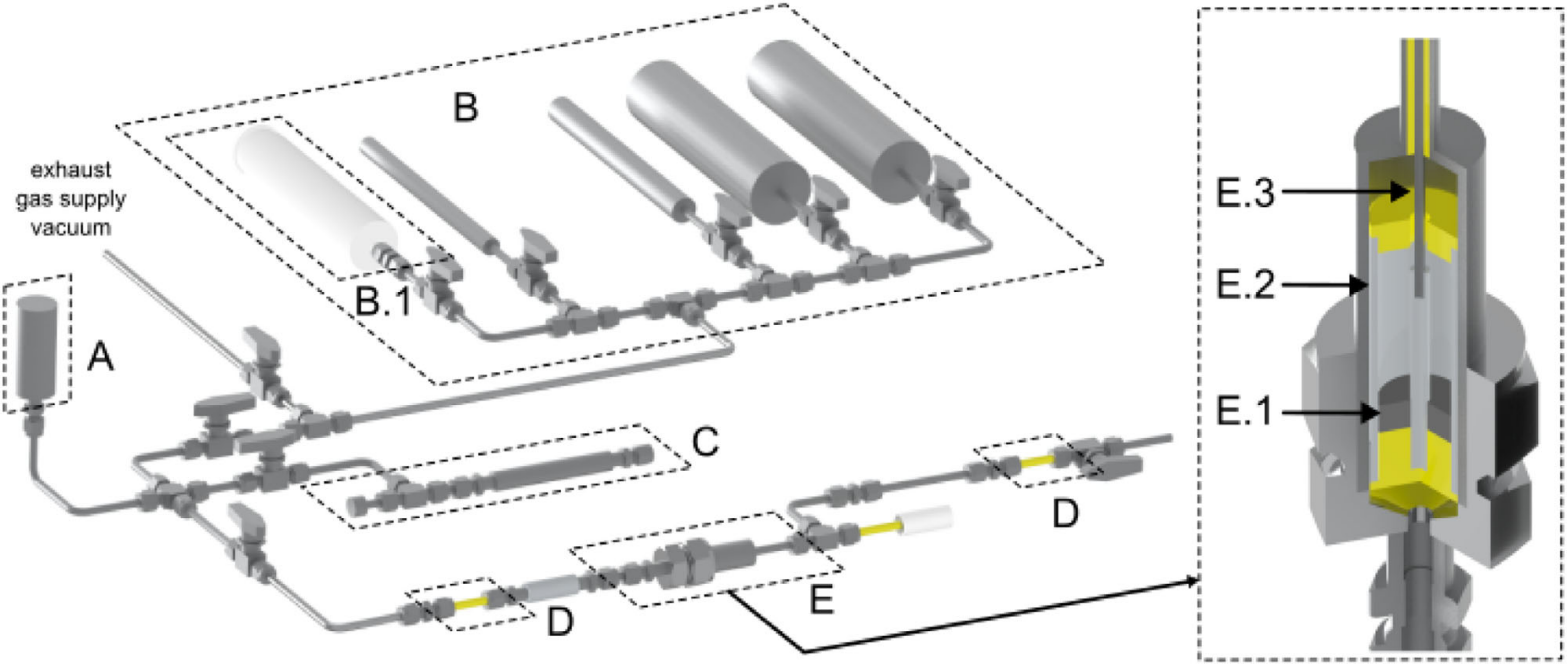
Experimental design for the adsorption/desorption swing experiments; pressure transducer A), volumetric measurement setup B) including gas piston prober (B.1), additional volume for volume adjustments C), PEEK‐tubes for electrical insulation D) and measurement cell E) including sample (E.1) as well as outer (E.2) and inner electrode (E.3); high pressure adsorption experiments where done in part E; gas was expanded to part B for volume measurement. (Design by Solid Edge©^[^ ^24^ ^]^).

For the electric field swing adsorption studies, a pressure volumetric setup was designed which ensures gas adsorption measurements using external electric fields under isothermal conditions (**Figure** **2**, for details of operation see Supporting Information).

**Figure 3:**
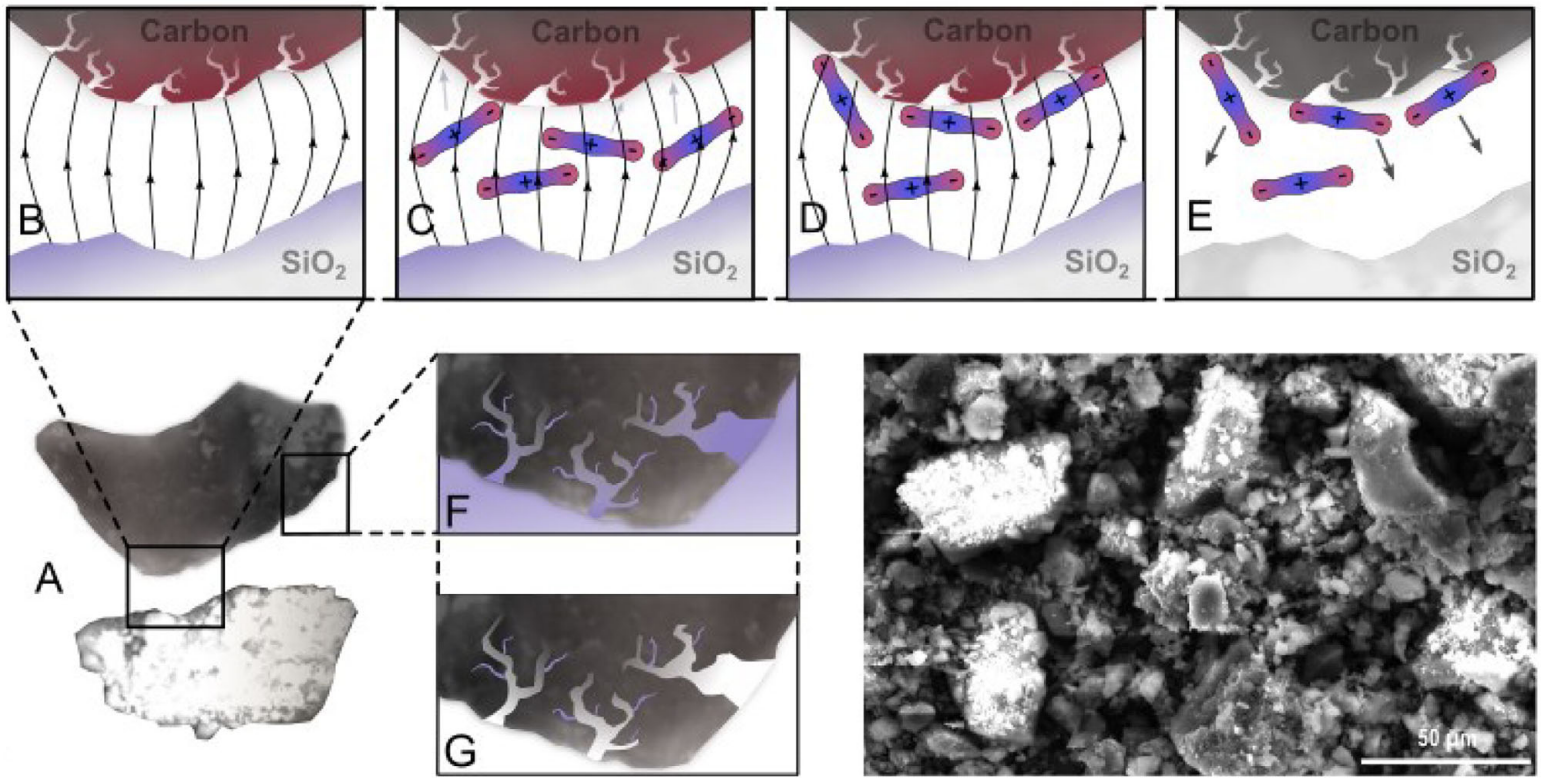
Cartoon type model for understanding swing adsorption process excess of CO_2_ at the solid/solid interface between an amorphous porous carbon (APC) and a SiO_2_ dielectric A) reflecting the interface situation of an APC/SiO_2_ composite particle. B) build‐up of the electrical field at the interface under vacuum. C) After pressure build up to 30 bar CO_2_ the gas molecules tend to orient in the electrical field and D) adsorb onto the carbon surface under isothermal conditions. E) After the field is switched off under constant pressure, the excess amount of CO_2_ gas due to electrical field adsorption is released and the adsorption/desorption swing process is finished leading to a pressure increase. F) Cartoon reflecting the porosity at the APC surface including G) filled micropores after gas removal. All drawings are not to scale, thus size dimensions are arbitary. Bottom right: HRSEM image of the virgin Norit/SiO_2_ composite (not metal sputtered). Thus, brighter spots are due to electrical charging in the SEM. The Norit/SiO_2_ mixture is homogenized by ball milling.

The selectivity of the adsorption is proven by the investigation of the excess adsorption of Ar, N_2,_ and CO_2_ at a pressure of ≈30 bar on the 1:1 Norit/SiO_2_ composite. The composition of the sample ensures a high number of conductor‐isolator interfaces. Such interfaces are known to store electrical energy by polarisation^[^ ^37^ ^]^(**Figure** **3**).

**Figure 4:**
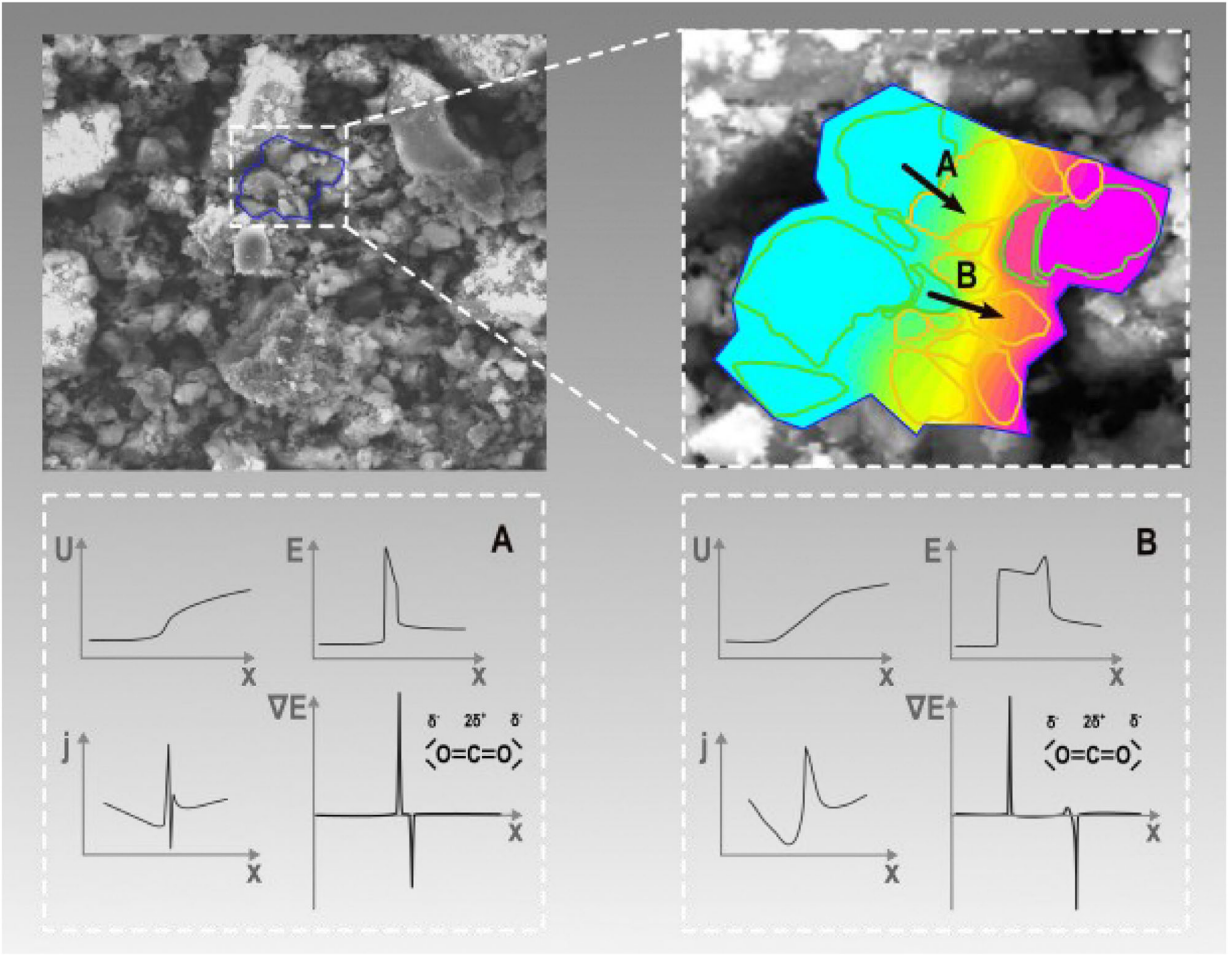
Top left: SEM‐picture of Norit‐SiO_2_ composite sample. Insert shows a randomly selected particle arrangement used for the calculation. Top right: Contour image of the calculated voltage‐ and current‐flow along A and B for the randomly selected particle ensemble of Norit/SiO_2_ (Norit: pale green; SiO_2_: dark yellow). Color coding denotes low (cyan) to high voltage (purple). Bottom left A) and right B): Electric potential (U), field strength (E), current density (j) and gradient of field strength (∇*E*). The curves show progressions across an ensemble of different Norit/SiO_2_ interfaces. Valence bond description showing the distribution of the quadrupole moment in CO_2_. Calculations done using FEMM 4.2^[^ ^44^ ^]^ see Section S2 (Supporting Information).

In addition to the existing dipole and quadrupoles of N_2_ and CO_2_, the application of an external field results in the formation of induced dipole‐ and quadrupole moments. These are mediated by the dipole–(α) and quadrupole (β) polarizabilities of the probe gases. The apparent dipole‐ (µ_α_) and quadrupole (µ_β_) moments are defined as a sum of the natural (µ_α,0_, µ_β,0_) and induced (µ_α,ind_, µ_β,ind_) multipoles by µ_α_ = µ_α,0_ + µ_α,ind_ and µ_β_ = µ_β,0_ + µ_β,ind_. The relations to the polarizabilities α and β are given by µ_α,ind_ = αE_local_ and µ_β,ind_ = β∇E_local_, where E_local_ is the local electric field and ∇E_local_ itsgradient. Using the coupling of the moments to the electric field based on a multipole description, the following equations regarding the corresponding energies U are valid

(1) $$U_{\alpha }=\mu_{\alpha ,0}E_{\mathrm{local}}+c_{\alpha }\;\alpha E_{\mathrm{local}}^{2}$$

(2) $$U_{\beta }=\mu_{\beta ,0}\nabla E_{\mathrm{local}}+c_{\beta }\;\beta {\left(\nabla E_{\mathrm{local}}\right)}^{2}$$

where c_α_ and c_β_ are numerical pre‐factors. As can be seen in Equations (1) and (2), the dipole‐related properties are connected to the local electric field. In contrast, quadrupole related properties are coupled to the gradient of the electric field. Electrical field and electrical field gradient describe two different aspects of possible local electric interactions. This is that the magnitude of the latter is much more restricted to the interface of particles as the former. As can be seen in **Figure** **4**, this local restriction expresses in a much more confined shape of ∇ E_local_ compared to E_local_ at the particular interfaces of the Norit/SiO_2_ individual particles.

The electric field E_local_ is influenced by inhomogeneities of the sample packing due to its non‐local effective area compared to ∇E_local_.^[^ ^37^ ^]^ Hence, in the macroscopic case, the dipole components of the gases play a major role. In the probe molecules Ar, N_2,_ and CO_2_ a dipole moment is only induced by the electric field, i.e., it is zero without the applied field. Microscopically, the apparent conductor‐isolator interfaces Norit versus SiO_2_ affect strong field gradients ∇ E_local_ due to the different conductivity and permittivity of these materials. As the quadrupole components are coupled to the field gradients (Equation (2)) located at the interfaces, the gas‐solid interaction related to this term should be strongly influenced by them. The differences in the quadrupole moments of Ar, N_2,_ and CO_2_ as shown in **Table** **1** suggest an inherent difference of their interaction with the applied electric field.

**Table 1:** Comparison of dipole‐ (µ_α,0_) and quadrupole (µ_β,0_) – moments as well as dipole‐ polarizability (α) and quadrupole‐ (β) polarizability of the gases Ar, N_2,_ and CO_2_.

gas	µ_α,0_	‐µ_β,0_ (10^−40^*Cm^2^)	α (cgs, Å^3^)	β (cgs,Å^5^)
Ar	0	0	1.641^[^ ^39^ ^]^	1.09^[^ ^40^ ^]^
N_2_	0	4.72^[^ ^38^ ^]^	1.740^[^ ^39^ ^]^	1.12^[^ ^41^ ^]^
CO_2_	0	13.4^[^ ^38^ ^]^	2.911^[^ ^39^ ^]^	3.32^[^ ^42^ ^]^

Another factor is the gas density ρ, which represents the number of gas molecules per volume element over a certain sample area. The experimental results are shown in **Table** **2**.

**Table 2:** Comparison of the experimental results. ρ_I_ denotes the sample resistance calculated from the applied voltage and the affected current, Δp_ΔU_ the pressure difference in the cell with and without electric field, n_ad,ΔU_ the amount of substance of gas adsorbed calculated from gas density difference related to Δp_ΔU_ and ΔV the volume difference measured; P was always ≈ 0.55 W.

gas	ρ_I_ (Ω)	Δp_ΔU_ (bar)	n_ad,ΔU (mmol)_	ΔV (ml)
Ar	893.53	0.335	0.225 ± 0.033	9.75 ± 1.59
N_2_	645.05	0.255	0.151 ± 0.041	9.1 ± 3.96
CO_2_	711.35	0.425	0.378 ± 0.107	16.2 ± 4.13

*n_ad,ΔU_* was calculated from the gas densities ρ at the given temperature and pressure from the NIST‐database^[^ ^43^ ^]^ under constant volume. ΔV was measured by expanding the excess gas into a volume equipped with a gas piston prober (see Figure 2).

### 2.2 Quantification of the EFSA Effect

**Figure 5:**
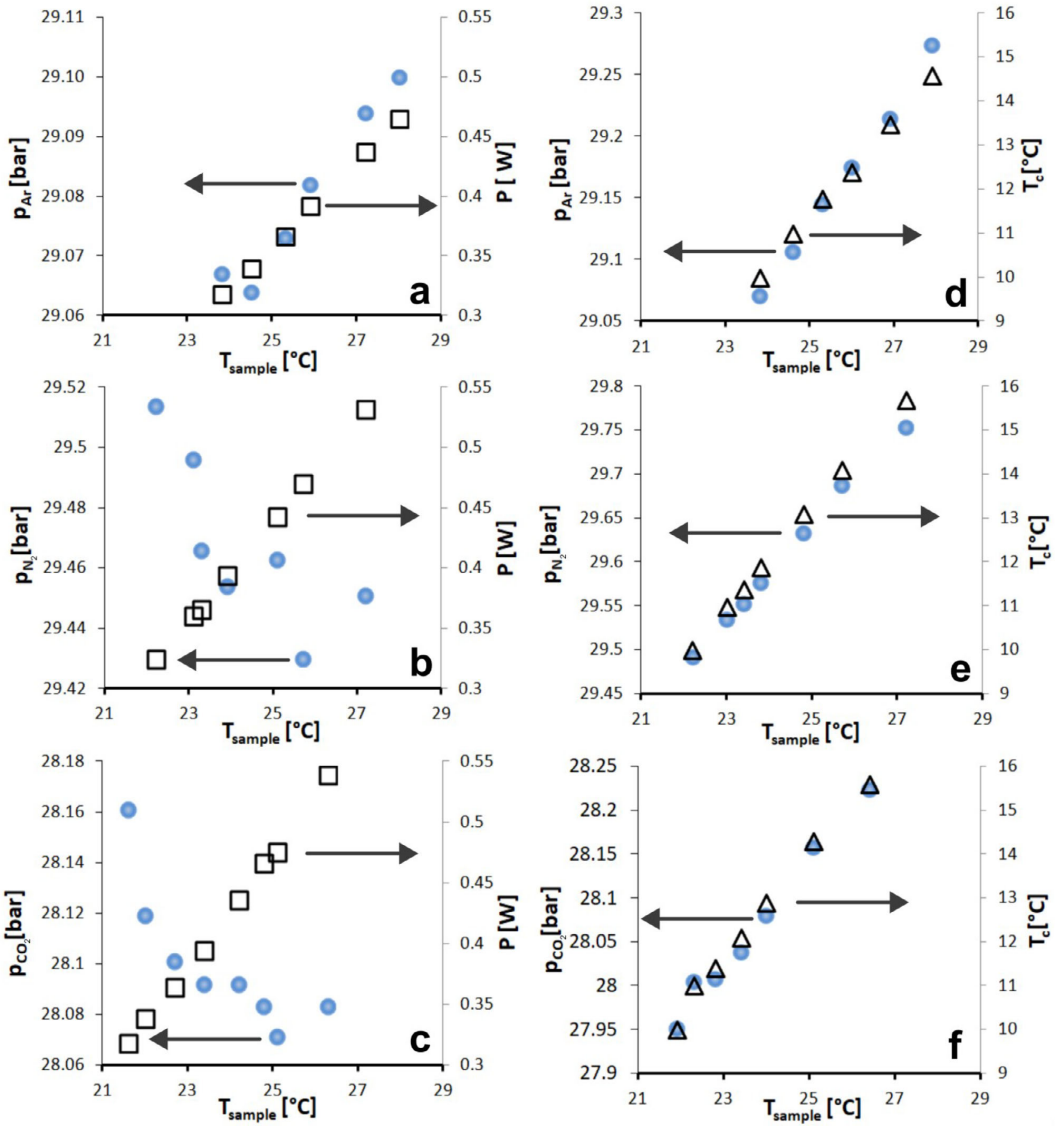
The Influence of P and T_c_ on adsorption characteristics is shown by p‐T_sample_‐curves (filled circles). An increase of P under constant T_c_ (upper figures a‐c; empty squares) results in slightly increasing (Argon; Figure 5.a) or decreasing (Nitrogen and Carbon Dioxide; Figure 5.b,c) pressure with increasing sample temperature. In contrast, an increase of T_c_ under constant P (lower figures d‐f; empty triangles) results in linear and increasing pressure with increasing sample temperature (0.1667 g of sample).

As the electrical field is applied, all carbon containing interfaces studied are influenced by the Joule‐effect. To compensate for this effect and to work under isothermal conditions an external cooling is applied. However, external cooling generates a temperature gradient within the sample cell and could be a potential source of a pressure decrease that masks the electrical swing adsorption effect. For this reason, we conducted two experimental campaigns in the high‐pressure regime. In the first, the counter‐cooling temperature *T_c_* was kept constant while the electrical power *P* was increased (**Figure** **5** a–c). In the second set, *T_c_* was increased while *P* was kept constant. Although the electrical field was not altered actively while keeping *P* constant (Figure 5d–f). The current slightly increased due to the increased temperature and gas pressure, respectively. Nevertheless, *P* was always in a very narrow range of 322 ±3 * 10^−3^ W in this set of experiments. The slopes of all *p‐T_sample_* – curves (filled circles) shown in Figure 5 estimated by Δp/ΔT_sample_ are shown in **Table** **3** (see Section S1.10, Supporting Information for fit functions).

**Table 3:** Slopes Δp/ΔT_sample_ (bar/°C) for all p‐T_sample_–curves shown in Figure 5 (filled circles) for constant counter cooling conditions and constant electric power.

Gas	*Δp/ΔT_sample_ (bar/°C) for c*onstant T_c_	*Δp/ΔT_sample_ (bar/°C) for* constant P
Ar	0.005	0.050
N_2_	−0.017	0.052
CO_2_	−0.020	0.063

Both conditions have a different influence on the p‐T_sample_ curves. In the case of constant electrical power, the pressure always increases linearly with the sample temperature showing slopes of 0.05–0.063 bar/°C. In contrast, constant counter cooling generates a much lower slope in case of Ar (0.005 bar/°C) compared to the former. Interestingly an even negative slope, corresponding to a decreasing pressure with increasing sample temperature is observed for N_2_ and CO_2_ (see also Figure 5b,c). This rather counter intuitive behaviour is however clearly due to the direct and now unshattered influence of the electric field which obviously is the source of pure excess adsorption of the Norit/SiO_2_ composite sample under the applied field.

The difference in the amount of substance Δn at 25 °C from this experimental set can be calculated by

(3) $$\Delta n=\left(n_{\mathrm{Tc},25^{\circ }C}-n_{P,25^{\circ }C}\right)\pm \left(n_{\mathrm{Tc},P/\mathrm{TStart}}-n_{P,p/\mathrm{TStart}}\right)$$

where the indices *P* and *T_c_* denote experiments with constant *P* and *T_c_*, respectively. The addition or subtraction of the correction term depends on the position of the starting points of the curves (see SectionsS1.10; S1.1.1 and S1.3.1, Supporting Information). Using the values given in the supplement (for volumes see Sections S1.1.1 and S1.3.1, Supporting Information; for densities see Table S9, Supporting Information) *Δn* can be calculated. **Table** **4** shows a comparison to n_ad,ΔU_.

**Table 4:** Comparison of the amount of substance of gas related to volume difference (n_ad,ΔU_) in constant P and T_c_ ‐experiments (Δn); Δ denotes the difference between Δn and n_ad,ΔU_.

Gas	Δn (mmol)	n_ad,ΔU_ (mmol)	Δ (mmol)
Ar	0.035	0.224	0.189
N_2_	0.107	0.150	0.043
CO_2_	0.303	0.377	0.074

As a result for all cases, it clearly shows that the application of an electrical field significantly generates an increase in gas adsorption. *n_ad,ΔU_* is in every case higher than *Δn*. The former value describes the difference between the on‐ and off‐state of the field at pressures considered for the three gases under study. The latter, *Δn* describes the difference between the influence of the two variables *T_c_* and *P*, which is the excess amount of gas adsorbed by applying the electric field. A comparison of the related adsorption capacity of CO_2_ to other electrified gas adsorption methods is shown in **Table** **5**.

**Table 5:** Comparison of excess adsorption capacity of CO_2_ of the electrified gas adsorption techniques EFSA, ESA, and MISA (magnetic field induced).

source	adsorption capacity (mmol/g)	technique	conditions	material
this work	1.88	EFSA	29 bar, 25 °C, 0.55 W	Norit RX 1.5 extra/SiO_2_
[15]	0.15	EFSA	1 bar, 0 °C, 800 V mm^−1^	K‐chabazite
[3]	1.0	ESA	1.6 bar, 30–60 °C	Maxsorb MSC‐30/Zeolithe 13 X
[45]	1.75	MISA	1.4 bar, 45 °C, 32 mT	MgFe_2_O_4_

To rule out any material changes in the composition of the Norit/SiO_2_ samples, BET, t‐plot, Raman spectrum and I_D_/I_G_‐ratio in Raman spectra reveal the compositional integrity of all samples after the full set of adsorption experiments (see Sections S1.5, S1.7.1 and S1.7.2, Supporting Information). A detailed discussion regarding the reproducibility and robustness of the results can be found in the Section S1.10 (Supporting Information).

To summarize, the difference between *Δn* and n_ad,ΔU_ (*Δ*; see Table 4) is a measure of the effectivity of the volumetric yield from excess adsorption. The excess amount of gas adsorbed by the electric field *Δn* is the lowest for Ar and increases for N_2_ by a factor of three and up to 10‐fold for CO_2_. These experimental results are in full accord with the significant increase in the quadrupole moments µ_β,0_ for Argon, N_2_ and CO_2_ as shown in Table 1.

## 3 Conclusion

It was shown that a moderate electric field can attract a considerable amount of surplus gas adsorption to a 1:1 sample of the amorphous carbon Norit and the dielectric SiO_2_ under isothermal conditions. This effect is dependent on the nature of the gas and its different polarizabilities and multipole distributions within the probe molecules which determine the interaction with the field. The applicability of excess adsorption in such a gas‐solid system controlled by a weak outer electric field was shown herein experimentally for the first time. The exploration of this effect might open the door for using other conductive species in connection with moderating dielectrics which are also prone to an extensive Joule heating effect in adsorption processes under electrical charging. On the other hand, notoriously non‐conductive materials, e.g., porous coordination compounds like MOFs, might become interesting for these processes when they are brought in intimate contact to a conductive phase.

For future investigations, the present approach of isothermal excess adsorption will be applied to mixtures of gases. In particular, the different extent of excess adsorption might be a possibility to induce a preferred interaction of a single gas component with the applied electric field. In a gas mixture, this might lead to a depletion of one component in the gas phase by a stronger interface related interaction. Such an approach points toward the possibility of the important target of gas separation at elevated pressures. Moreover, the use of hybrid materials with a well‐designed dielectric shell on a carbonic material to control the overall resistance of the bulk material and to optimize the conductor‐resistor‐interface might be helpful to further exploiting the investigated effect. Investigations of the interface controlled electrical swing absorption effect in lower pressure regimes might help to increase the applicable range of the technique and further prove its general validity. Carbonyl sulfide seems to be an interesting candidate, however, which liquefies at much lower pressures^[^ ^43^ ^]^ but shows no chemisorption^[^ ^46^ ^]^ while having a strong dipole moment^[^ ^47^, ^48^ ^]^ but a significantly lower quadrupole moment.^[^ ^48^ ^]^

## Conflict of Interest

The authors declare no conflict of interest.

## Supporting information



Supporting Information

## Data Availability

The data that support the findings of this study are available in the supplementary material of this article.
