# Supplementary material for: Interface Controlled Electric Field Swing Adsorption
Source: Adv Sci (Weinh). 2025 Jul 3;12(36):e04617. doi: 10.1002/advs.202504617 (PMC12463098; doi:10.1002/advs.202504617)
Supplement: Supplementary file 1 — Supporting Information [file ADVS-12-e04617-s001.pdf]

ADVANCED SCIENCE
Open Access

Supporting Information

for *Adv. Sci.*, DOI 10.1002/advs.202504617

Interface Controlled Electric Field Swing Adsorption

*Silvio Heinschke and Jörg J. Schneider**

Interface Controlled Electric Field Swing Adsorption - Supplementary

Silvio Heinschke and Jörg J. Schneider *

Technische Universität Darmstadt, Darmstadt, Germany, Eduard-Zintl-Institut für Anorganische und Physikalische Chemie, Peter-Grünberg-Str. 12

E-mail: joerg.schneider@tu-darmstadt.de

Phone: +49 (0)6151 1621100. Fax: +49 (0)6151 1621102

Contents

1 Experimental section **3**

1.1 Setup 3

1.1.1 Calibration 4

1.1.2 Sample cell 6

1.2 Sample preparation, adsorption experiments and related information 6

1.3 Measurement of excess volume 8

1.3.1 High pressure regime 8

1.3.2 Low pressure regime 9

1.4 Repeatability 9

1.5 Raman measurements 9

1.6 Calculation of specific resistance 11

1.7 Data 11

1.7.1 Raman 11

1.7.2 Nitrogen-isotherms 11

1.7.3 Experiments 13

1.8 Accuracy and handling of the gas piston prober 13

1.9 Data calculation 17

1.10 Reproducibility of the EFSA-effect 19

2 Simulation of voltage and current flow **20**

References **21**

1 Experimental section

1.1 Setup

The setup is build out of stainless-steel tubes (1.4301) and parts connected by Swagelok® fittings. The system consists of two temperature sensors (K-type, Omega Engineering limited) and a pressure sensor (PAA33X-V-30, 0-30bar, Omega Engineering Limited, accuracy (ability of the sensor to recognize a pressure difference): -0.002 to 0.001% from Maximum value (30 bar; -0.6 to 0.3 mbar), digital overall error (including measurement uncertainty and temperature error; digital signal rs4 port): 0.05% from Maximum value (30 bar; +- 30 mbar); equipped with a Digital Multimeter (Model 3360, Peaktech) is used to determine the pressure). The sample chamber was isolated using PEEK-tubes with with $6mm$ outer diameter and $1.65mm$ inner diameter which where connected to the system by Swagelok® fittings. The wall of the sample chamber was used as outer electrode. It was isolated from the inner electrode by PEEK-tubes. A Sorensen® XEL 250 DC Power Supply was used to apply the electric field to the sample. The system tested leakage free using He (He 4.8, Air liquide). The temperature is measured directly at the sample with a K-type NiCr thermocouple (OMEGA Engineering Inc.). The desired temperature is adjusted and equilibrated with a air ventilation based thermoregulatory system connected to a cryostat (Haake Fisons R). The difference in gas volume was measured by a self-build external device equipped with a gas piston prober (Roth) with a maximum volume of 50 ml.
A simplified drawing of the system is shown in fig. 1.

The volumes, temperatures and pressure values corresponding to fig. 1 are: sample chamber (V_{SC},T_{SC},p_{SC}), Volume 1 (V_1,T_1,p_1), Volume 2 (V_2,T_2,p_2), section H (V_H,T_H,p_H), section L (V_L,T_L,p_L), gas piston prober (V_P,p_P). The small volume which can be found between the valves (including the pressure transducer) is defined as $V_H - V_{SC} - V_1 = V_s$ (T_s,p_s).
The whole system was included into an isolated housing. Inside the housing, a system of

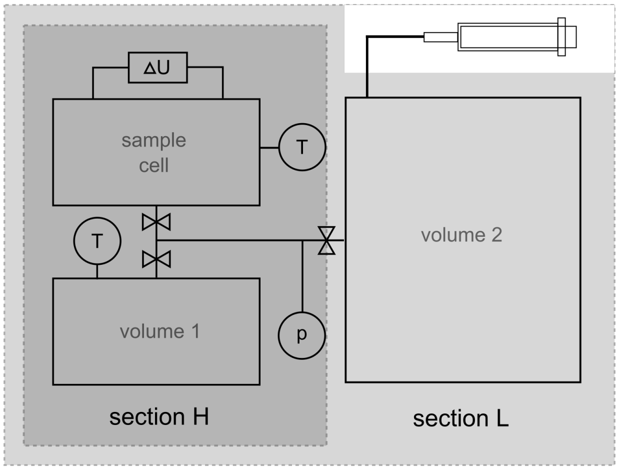

Figure 1: system overview

tubes connected to cryostats (Julabo F25, Haake K/F3) are installed. The heat exchange was conducted by several fans which where oriented towards the actual adsorption chambers. A comparable system can be found e.g. at Möllmer[1]

1.1.1 Calibration

The calibration of volume 1 with N_2-gas ($N_2 5.0$, Air liquide) was accomplished by the method described by Möllmer.[1] Herein, a block of OFHC-Copper (purity > 99.99 %) with known mass ($m_{Cu-block} = 21.11215g$, $\rho_{Cu} = 8.96\frac{g}{cm^3}$,[2] $VCu - block, calc = 2.36cm^3$) was inserted into V_{SC}. Hereafter, the equilibrium values of pressure ($\approx 18bar$) and temperature ($\approx 25°$) in $V_{SC} + V_s$ where measured. In the following, the gas was expanded expanded into $V_{SC} + V_s + V_1$ and the equilibrium values where noted again. Additionally, the measurements where conducted without Cu-block in V_{SC}. The calibration values are given in the tables 1 and 2. Herein, V_B is an extra volume only used to calibrate V_2. Hence, it is not included in figure 1. Other volumes than V_1 where calculated by $\left[\frac{pV}{T}\right]_x = \left[\frac{pV}{T}\right]_y$, where x and y label specific volumes. The value of V_{SC} was determined twice as it was necessary to interchange the sample cell between the experiments.

The following values resulted (mean values including standard deviation): $V_1 = 17.45 \pm 0.43ml$, $V_s = 16.73 \pm 0.02ml$, $V_{SC} = 16.43 \pm 0.01ml$, $V_B = 17.21 \pm 0.01ml$ and $V_2 =$

Table 1: calibration values before expansion

calibration V_1	$T_{SC}\,(^\circ C)$	p in $V_{SC}+V_s\,(bar)$	ρ_{N_2} at $T_{SC}\,\left(\frac{g}{cm^3}\right)$
with Cu-block	25.2	18.518	0.0364
	25.2	18.474	0.0363
	25.2	18.525	0.0365
without Cu-block	25.2	18.315	0.0360
	25.1	18.366	0.0361
	25.1	18.405	0.0362
calibration V_s	$T_1\,(^\circ C)$	$p_1\,(bar)$	
	25.0	18.138	
	25.0	18.525	
	25.1	18.609	
V_{SC}	$T_1\,(^\circ C)$	$p_s\,(bar)$	$T_{SC}\,(^\circ C)$
	24.9	18.075	24.9
	25.0	18.018	24.9
	24.9	18.114	24.9
calibration V_B	$T_1\,(^\circ C)$	$p_s\,(bar)$	$T_B\,(^\circ C)$
	25.1	18.066	24.9
	25.0	18.066	25.0
	24.9	18.234	24.9
calibration V_2	$T_1\,(^\circ C)$	$T_{SC}\,(^\circ C)$	p in $V_s+V_1+V_{SC}+V_B\,(bar)$
	24.7	24.6	28.358
	24.7	24.8	28.425
	24.8	24.8	28.401

I

Table 2: calibration values after expansion

calibration V_1	$T_{SC}\,(^\circ C)$	$T_1\,(^\circ C)$	p in $V_{SC}+V_s+V_1\,(bar)$	ρ_{N_2} at $T_{SC}\,\left(\frac{g}{cm^3}\right)$	ρ_{N_2} at $T_1\,\left(\frac{g}{cm^3}\right)$	$\frac{\rho_{V_{SC}+V_s}}{\rho_{V_{SC}+V_s+V_1}}$
with Cu-block	25.2	25.0	12.399	0.0235	0.0235	1.549
	25.1	25.1	12.3645	0.0235	0.0235	1.545
	25.2	25.1	12.4005	0.0235	0.0235	1.553
without Cu-block	24.9	24.9	12.543	0.0238	0.0238	1.512
	25.1	25.1	12.576	0.0239	0.0239	1.510
	25.0	25.1	12.606	0.0240	0.0239	1.515
calibration V_s	p in $V_s+V_1\,(bar)$					
	9.213					
	9.400					
	9.444					
V_{SC}	p in $V_s+V_{SC}\,(bar)$					
	9.108					
	9.081					
	9.135					
calibration V_B	p in $V_s+V_B\,(bar)$					
	8.902					
	8.906					
	8.988					
calibration V_2	$T_1\,(^\circ C)$	$T_{SC}\,(^\circ C)$	p in $V_s+V_1+V_{SC}+V_B+V_2\,(bar)$			
	24.8	24.7	1.729			
	24.8	24.7	1.733			
	24.9	24.9	1.733			

$1044.87 \pm 0.28 ml$. Density values where taken from the NIST-database.[3]

1.1.2 Sample cell

The design of the sample cell is shown in figure 2.

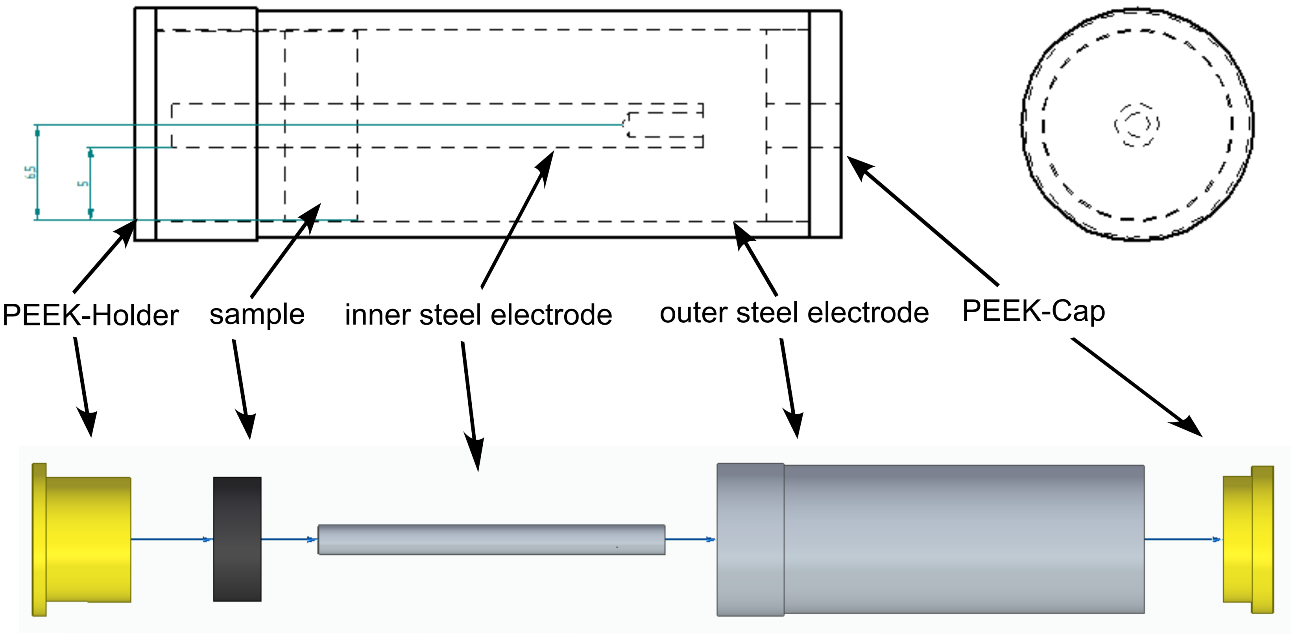

Figure 2: sample cell including radii in mm

1.2 Sample preparation, adsorption experiments and related information

Activated carbon Norit RX 1.5 Extra©; Cabot Norit Nederland B.V.; extruded was grinded in a ball mill (Fritsch; Pulverisette 23) at 50 Hz for 10 min. The grinded activated carbon was boiled three times under reflux in distilled water, isopropanol and diethyl ether and subsequently degassed under vacuum at 150 °C and flushed with argon afterwards. The procedure was repeated for SiO2 (Kieselgel 60; Macherey-Nagel GmbH& Co. KG; 0.2 - 0.5 µm). The appropriate masses of activated carbon and SiO2 (0.787 and 0.790g) where intimately mixed in a ball mill (Pulverisette 23, Fritsch GmbH) at 50 Hz for 10 min. This

1:1 by weight composite was used for the absorption experiments. High resolution scanning electron microscopy (HRSEM) investigations were carried out by using a Philips XL-30 FEG operated at 15 kV and 10 mm working distance. The samples were placed on conductive Carbon tape and sputtered with 3 nm Pt/Pd (80/20) using a Cressington HR 208 sputter coater. Gases (Ar Arcal Prime, N2 N50, CO2 N45) where purchased from Air Liquide.

Before experiment, the sample was filled into the sample cell (see 2) and slightly compressed with a plastic stamp at $10\frac{kg}{cm^2}$. The weight difference between the empty cell and the cell including the compressed sample was taken as the actual sample weight. Sample height was determined. Afterwards, the sample cell was installed into the system. The sample was degassed prior to experimental series in vacuum at $150°C$ for $24h$ to remove adsorbed gases and water. Between each measurements the samples were degassed over night (approximately $16h$) under vacuum at $25°C$. The influence of electrical power P and counter cooling temperature T_c was investigated. During the volumetric experiments, P was kept constant. Four measurements for each probe gas for adsorption experiments with and without electric field were carried out. In the latter, the electric field was applied on a vacuumed sample followed by subsequent purge of the probe gas. The difference in pressure and volume with and without applied field under equilibrium conditions was measured. The experimental procedure to load the sample with gas with and without applied electric field is explained in the following:

After the closed sample chamber with the degassed sample had been installed in the body of the system, the entire system was evacuated and the sample temperature was set to the desired value by adjusting the temperature of the thermoregulator to the required value. After the temperature was set, if needed, the electrical potential was applied at the sample while it was still under vacuum. The conductivity of the sample under vacuum turned out to be higher then under application of a gas, which is comparable to the literature.[4,5] As a result, the sample temperature as well as the current increased immediately. After setting the appropriate voltage, a pressure of $\approx$ 30 bar using the desired gas was applied in the

section H of the setup (see fig. 1), which stabilizes current and temperature. If necessary, the voltage was altered to achieve the desired electrical power applied to the sample. After this procedure, equilibration of the temperature was ensured. In case of an applied electrical potential, it was switched off and the system was equilibrated. The corresponding values are denoted as $p_{\Delta U}$ and $p_{\Delta U=0}$. The difference is assigned to the amount of gas $n_{ad,\Delta U}$. For volumetric experiments, the pressure was released into section L of the system (p_L) (see fig. 1). After another equilibration of the temperature, the valve to the gas piston prober was opened. Herein, the parameter $V_{P,calc,\Delta U}$ (mL) represents the Volume measured by the gas piston prober calculated from a calibration curve. Details regarding this specific issue can be found in chapter 1.8.

1.3 Measurement of excess volume

1.3.1 High pressure regime

Table 3: results for 0.1613g of sample at high pressure

gas	$T_{\Delta U}$ ($^\circ C$)	$p_{\Delta U}$ (bar)	$\rho_{\Delta U}$ $\left(\frac{g}{ml}\right)$	$T_{\Delta U=0}$ ($^\circ C$)	$p_{\Delta U=0}$ (bar)	$\rho_{\Delta U=0}$ $\left(\frac{g}{ml}\right)$	$n_{\Delta U}$ $(mmol)$	$n_{ad,\Delta U}$ $(mmol)$	$\frac{n_{ad,\Delta U}}{n_{\Delta U}}$ (%)
Ar	25.3	29.100	0.047655	24.6	29.445	0.048353	19.388	0.258	1.33
Ar	24.9	29.103	0.047730	24.7	29.478	0.048390	19.418	0.244	1.23
Ar	24.8	29.040	0.047642	25.0	29.391	0.048192	19.382	0.203	1.05
Ar	25.1	29.103	0.047695	24.7	29.370	0.048210	19.404	0.189	0.98
N_2	25.1	29.589	0.033554	25.2	29.880	0.033872	19.467	0.184	0.95
N_2	24.9	29.526	0.033506	25.3	29.733	0.033693	19.439	0.109	0.56
N_2	25.2	29.571	0.033521	25.3	29.865	0.033843	19.448	0.187	0.96
N_2	24.9	29.553	0.033537	25.3	29.781	0.033748	19.457	0.123	0.63
CO_2	24.7	28.167	0.059433	25.2	28.506	0.060127	21.948	0.256	1.17
CO_2	25.1	28.257	0.059524	25.1	28.617	0.060448	21.981	0.339	1.54
CO_2	24.5	28.206	0.059604	24.7	28.770	0.060987	22.011	0.511	2.32
CO_2	25.1	28.242	0.059486	25.2	28.677	0.060567	21.967	0.399	1.82

$n_{ad,\Delta U} = (V_{SC} - V_{sample})\,\frac{\rho_{\Delta U=0}-\rho_{\Delta U}}{M_{gas}}$ and $n_{\Delta U} = (V_{SC} - V_{sample})\,\frac{\rho_{\Delta U}}{M_{gas}}$ are calculated from the gas densities ρ at the given temperature T from the NIST-database.[3] Here, $V_{SC} = 16.43ml$ denotes the sample cell volume and $V_{sample} = \pi r_o^2 h_{sample}$ the sample volume with $r_o = 0.5cm$ and $r_i = 0.15mm$ being the difference between the radii of the outer and inner electrode of the cell and the radius of the inner electrode (see also 2). The sample height is $h_{sample} = h_{S_2} = 0.225cm$. Hence the sample volume is $V_S = 0.177cm^3$. The single pressure regimes for the gases where chosen under the aspect that $p_{\Delta U=0}$ doesn't exceed the tolerance

of the pressure transducer ($\leq 30bar$) and that the resulting volume difference at low pressures is measurable with the gas prober.

1.3.2 Low pressure regime

The Volume differences are calculated from a calibration curve (further explanation is given in chapter 1.8).

Table 4: results for 0.1613g of sample

gas	$P\left(10^3W\right)$	$\rho\,(\Omega cm)$	$\frac{n_{ad,\Delta U}}{n_{\Delta U}}$ (%)	$V_{P,calc}\,(mL)$	$V_{P,calc,\Delta U}\,(mL)$	$\Delta V\,(mL)$
Ar	553.3	884.4	1.33	36.9	45.8	8.9
Ar	549.4	904.1	1.23	36.9	44.9	8.0
Ar	550.6	888.7	1.05	36.0	47.5	11.5
Ar	545.3	897.4	0.98	37.8	48.4	10.6
N_2	552.1	668.4	0.95	26.2	39.6	13.4
N_2	549.7	665.5	0.56	24.5	36.0	11.5
N_2	549.2	643.2	0.96	31.6	37.8	6.2
N_2	545.0	603.1	0.63	31.6	36.9	5.3
CO_2	546.1	585.4	1.17	21.8	34.2	12.4
CO_2	542.1	747.2	1.54	16.5	29.8	13.3
CO_2	549.2	743.6	2.32	15.6	33.3	17.7
CO_2	551.0	759.5	1.82	20.0	41.3	21.3

1.4 Repeatability

The repeatability of the effect of the electric field was investigated. Therefore, the sample was loaded with Ar up to a pressure of $\approx$ 29.1 bar under charging at a sample equilibration temperature of $\approx 25°C$. The field was switched off and Δp was measured after equilibration. Thereafter, the field was switched on at the pressure achieved and the process was repeated.

Herein, both on-off states showed a pressure difference $\approx 0.45bar$.

1.5 Raman measurements

The Raman-spectra of the samples are plotted in fig. 4 show several characteristics. Besides D- and G-Band, the signal at $\approx 2650cm^{-1}$ can be assigned the 2D-mode,[6–8] which is related to overall ordering of the structure.[8] Its rather broad appearance indicates the absence of ordering in the samples, which is comparable to the literature.[8] Furthermore, the signals at $\approx 1032cm^{-1}$, $\approx 2895cm^{-1}$ and $\approx 3172cm^{-1}$ can be assigned to the D”-, D+G- and 2D’-mode.[7] The signals at $\approx$ 454 and $\approx 839cm^{-1}$ might be associated with an amorphous and

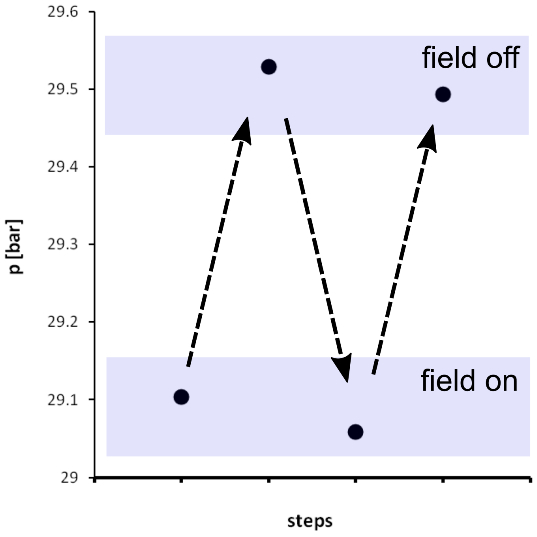

Figure 3: On-off experiment using Ar

graphitized structural part of the sample. A hint towards this interpretation can be found in Tan et al.[7] and Kawashima et al.[9] In the former, signals in this range are associated with the density of states of C^+-ion implanted highly ordered pyrolytic graphite (632.8nm laser wavelength). In the latter, a signal at 803, 810 and 820cm^{-1} was found using laser wavelengths of 457.9, 488 and 514.5nm in a pyrolytic graphite sample which gives rise to reasonable shift to higher wavenumbers using a 532nm laser. In the additionally investigated highly ordered pyrolytic graphite, this signal mostly disappeared. Along with this signal, Kawashima et al.[9] showed the existence of a signal at 1071, 1083 and 1094cm^{-1} using 457.9, 488 and 514.5nm laser wavelength. In our samples a comparable peak can be found at $\approx$ 1040cm^{-1}. However, a possible interpretation about its origin can be found in Fantini et al.,[10] where it is associated to possible $C-O-C$-features of the sample. Moreover, the 840cm^{-1}-Peak (860cm^{-1},845cm^{-1}[10]) is associated with the occurrence of graphitic structures herein. In general, the sample before and after the experiments show no visible changes regarding peak appearances or heights. SiO_2 showed no characteristic Raman-Signals. The signal at 2300cm^{-1} is associated to athmospheric N_2.

Individual Raman D-Band ($\approx$ 1330 cm-1) to G-Band ($\approx$ 1577 cm-1) ratios (ID/IG) of the activated carbon, the composite sample before and after swing adsorption experiments were

fitted using a Gauß-Lorenz-fit resulting in values ID/IG of 2.25 and 2.26, respectively.

1.6 Calculation of specific resistance

The specific resistance in the sample cell is calculated by[11]

$$\rho = R\left[\frac{1}{2\pi h_{sample}}\int_{r_i}^{r_o}\frac{dr}{r}\right]^{-1} = R\frac{2\pi h_{sample}}{ln\frac{r_o}{r_i}} \quad (1)$$

where h_{sample} is the sample height, r_o the inner radius of the outer electrode and r_i the outer radius of the inner radius.

1.7 Data

1.7.1 Raman

Raman measurements were carried out with a DXR3 Raman microscope (Thermo Fisher) using a wavelength of 532 nm 200 and 350 cm-1. Spectra were analysed using OMNIC-Software (Thermo-Fisher).
Data of Raman-measurements is shown in fig. 4.

1.7.2 Nitrogen-isotherms

Nitrogen adsorption isotherms measurements were carried out in a NOVA 3000e (Quantachrome) at 77 K using liquid nitrogen from 5*10-3 to 0.3 p/p0. Samples where degassed at 150°C for 12 h prior to measurement. Brunauer-Emmett-Teller (BET) method was used to determine the specific surfaces of the samples. Micropore and external specific surface area where calculated using t-plot method.
1:1 composite sample before and after adsorption experiment show a type IV-isotherm (see fig. 5). The isotherms for activated carbon and SiO_2 are shown in fig. 6 and 7. BET and t-plot results are shown in table 5.

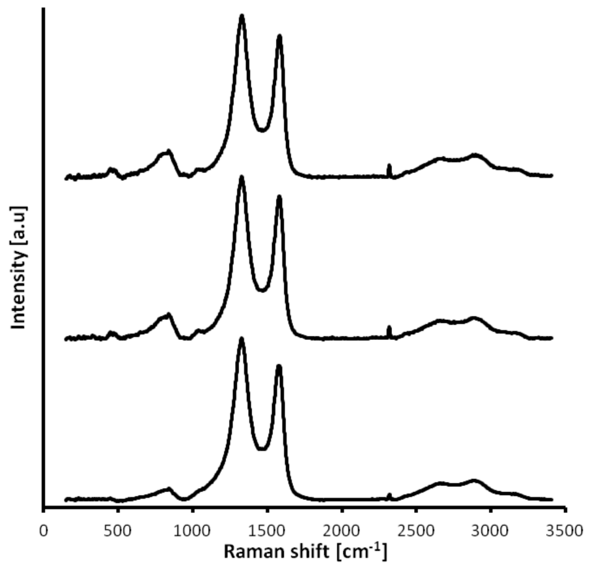

Figure 4: Raman spectra of amorphous carbon NoritR, a 1:1 composite sample before and after swing adsorption (from bottom to top, average spectra, measured at 10 different sample spots each)

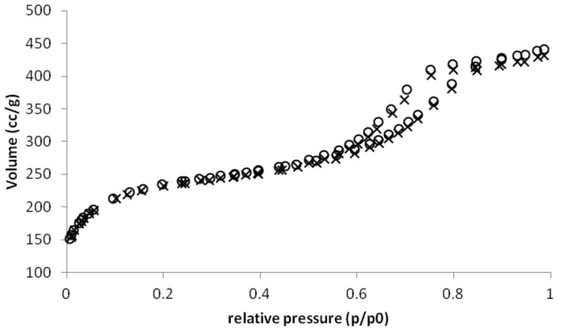

Figure 5: Isotherms of an 1:1 composite NoritR/SiO2 composite sample before (open circles) and after (crosses) volume expansion experiments

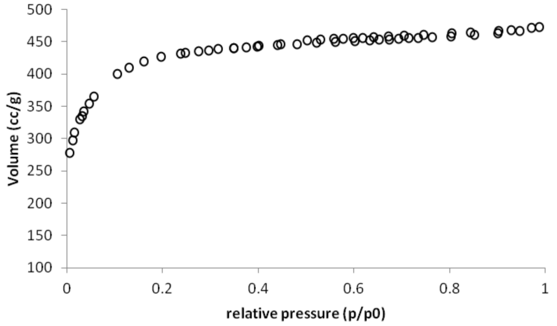

Figure 6: isotherm of activated carbon (Norit RX1.5)

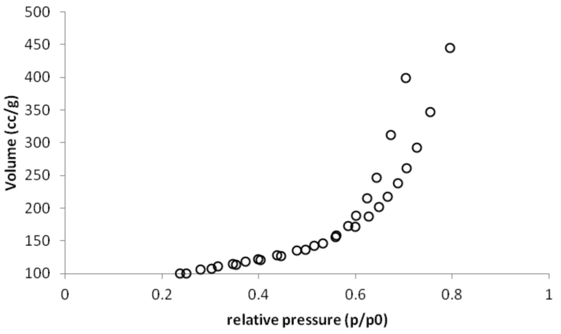

Figure 7: isotherm of SiO_2 (Kieselgel 60 $0.2 - 0.5$µm)

Table 5: Summary of BET and t-plot results of single activated carbon and SiO2, a composite sample and a composite sample after measurement

sample	BET-surface $\frac{m^2}{g}$	Micropore area $\frac{m^2}{g}$	External surface area $\frac{m^2}{g}$
amorphous carbon	1473.9	1156.3	317.6
SiO2	339.4	28.1	311.3
1:1 composite sample before experiments	801.2	473.8	327.4
1:1 composite sample after experiments	797.3	477.8	319.5

1.7.3 Experiments

Experimental parameters and results are shown in table 6, 7 and 8.

1.8 Accuracy and handling of the gas piston prober

The gas piston prober is connected to the main body of the experimental setup by screw connector sealed with PEEK (Polyether ether ketone) and Viton® rings. The gas from the setup volume is purged into the prober by manually opening the related valve. A calibration curve (V vs. p) was measured for N_2. The data can be found in table 9.

The values given in table 9 can be fitted by the linear function

$$V_{N_2} = 887.71 * p - 902.31 \tag{2}$$

with a coefficient of determination of 0.9842.

The actual volumes measured by the gas piston prober highly depend on the difference between p_L and the atmospheric pressure. The latter is slightly changing from values of $0.98 bar$ and $1.01 bar$. We relate every measurement to the parameter $V_{P,calc,\Delta U}$ (mL) which

Table 6: experimental parameters

	Experiments without electrical field					Experiments with electrical field									
m_{sample} (mg)	p_1 (bar)	p_{SC} (bar)	p_H (bar)	p_L (bar)	V_P (mL)	I (mA)	U (V)	p_1 (bar)	$p_{SC,\Delta U}$ (bar)	$p_{SC,\Delta U=0}$ (bar)	$\Delta p_{\Delta U}$ (bar)	Δt $(min)^{I}$	p_H (bar)	p_L (bar)	V_P (mL)
0.1613	9.942	29.139	22.608	1.058	38.5	20.7	26.6	9.890	29.040	29.391	0.351	52	22.773	1.068	≈ 54.0
0.1613	9.859	29.106	22.554	1.058	33.0	20.5	26.6	9.864	29.103	29.370	0.267	22	22.776	1.067	≈ 54.0
0.1613	9.852	29.064	22.572	1.057	26.5	20.8	26.6	9.9018	29.1	29.445	0.345	20	22.842	1.070	49.0
0.1613	9.858	29.139	22.617	1.059	32.0	20.5	26.8	9.891	29.103	29.478	0.375	53	22.866	1.071	50.0
0.1613	9.5493	29.601	22.575	1.046	24.0	23.9	23.1	9.3435	29.589	29.880	0.291	94	22.890	1.061	22.5
0.1613	9.4056	29.622	22.488	1.044	28.0	23.9	23.0	9.3351	29.526	29.733	0.207	38	22.815	1.057	28.0
0.1613	9.3723	29.550	22.680	1.052	33.5	24.3	22.6	9.3426	29.571	29.865	0.294	62	22.851	1.059	30.0
0.1613	9.3162	29.601	22.692	1.052	28.0	25.0	21.8	9.351	29.553	29.781	0.228	161	22.773	1.058	38.5
0.1613	0	28.278	19.797	1.041	21.5	22.4	24.6	0	28.242	28.677	0.435	58	20.112	1.055	35.5
0.1613	0	28.254	19.776	1.035	21	25.4	21.5	0	28.167	28.506	0.339	20	19.992	1.050	33
0.1613	0	28.194	19.746	1.034	19	22.4	24.2	0	28.257	28.617	0.360	75	20.091	1.054	27.5
0.1613	0	28.224	19.761	1.039	24.5	22.6	24.3	0	28.206	28.770	0.564	61	20.238	1.063	43.0

on time of electrical power

Table 7: equilibrium temperatures of experiments related to table 6

		Experiments without electrical field					Experiments with electrical field							
gas	$m_{sample}\,(mg)$	$T_1\,(^\circ C)$	$T_{SC}\,(^\circ C)$	$T_H\,(^\circ C)$	$T_L\,(^\circ C)$	$V_P\,(mL)$	$I\,(mA)$	$U\,(V)$	$T_1\,(^\circ C)$	$T_{SC,\Delta U}\,(^\circ C)$	$T_{SC,\Delta U=0}\,(^\circ C)$	$T_H\,(^\circ C)$	$T_L\,(^\circ C)$	$V_P\,(mL)$
Ar	0.1613	25.2	25.2	25.3	25.1	38.5	20.7	26.6	24.9	24.8	25.0	25.1	25.1	≈ 54.0
Ar	0.1613	25.3	25.1	25.1	25.2	33.0	20.5	26.6	24.7	24.9	24.7	24.9	25.0	≈ 54.0
Ar	0.1613	24.6	24.6	24.6	24.7	26.5	20.8	26.6	24.8	25.3	24.6	25.1	24.9	49.0
Ar	0.1613	24.7	24.7	24.8	24.8	32.0	20.5	26.8	24.7	24.9	24.7	24.9	25.0	50.0
N_2	0.1613	24.9	24.9	25.0	25.0	24.0	23.9	23.1	25.0	25.0	25.1	25.2	25.2	22.5
N_2	0.1613	24.9	25.1	25.0	25.1	28.0	23.9	23.0	25.2	25.3	24.9	25.7	25.0	28.0
N_2	0.1613	24.8	24.8	25.0	24.9	33.5	24.3	22.6	25.1	25.2	25.3	25.5	25.3	30.0
N_2	0.1613	25.0	25.1	25.1	25.1	28.0	25.0	21.8	24.9	24.9	25.3	25.2	25.2	38.5
CO_2	0.1613	–	25.1	25.2	25.3	21.5	22.4	24.6	–	25.1	25.2	24.9	24.9	35.5
CO_2	0.1613	–	24.7	24.8	25.1	21	25.4	21.5	–	24.7	25.2	25.2	25.1	33
CO_2	0.1613	–	24.9	25.3	25.1	19	22.4	24.2	–	25.1	25.1	24.9	25.1	27.5
CO_2	0.1613	–	25.0	24.9	25.2	24.5	22.6	24.3	–	24.5	24.7	25.3	25.0	43

Table 8: results of experiments

gas	$h_{sample}\,(cm)$	$m_{sample}\,(mg)$	$t\,(min)$	$p_{SC,\Delta U}\,(bar)$	$p_{SC,\Delta U=0}\,(bar)$	$p_L\,(bar)$	$V_P\,(mL)$	$V_{P,calc}\,(mL)$	$\rho\,(\Omega cm)$	$P\left(10^3 W\right)$
Ar	0.225	0.1613	–	–	29.139	1.058	38.5	36.9	–	–
	0.225	0.1613	–	–	29.106	1.058	33.0	36.9	–	–
	0.225	0.1613	–	–	29.064	1.057	26.5	36.0	–	–
	0.225	0.1613	–	–	29.139	1.059	32.0	37.8	–	–
	0.225	0.1613	20	29.100	29.445	1.070	49.0	47.5	884.4	553.28
	0.225	0.1613	53	29.103	29.478	1.071	50.0	48.4	904.1	549.40
	0.225	0.1613	52	29.040	29.391	1.068	≈ 54.0	45.8	888.7	550.62
	0.225	0.1613	22	29.103	29.370	1.067	49.0	44.9	897.4	545.30
N_2	0.225	0.1613	–	–	29.601	1.046	24.0	26.2	–	–
	0.225	0.1613	–	–	29.622	1.044	28.0	24.5	–	–
	0.225	0.1613	–	–	29.550	1.052	33.5	31.6	–	–
	0.225	0.1613	–	–	29.601	1.052	28.0	31.6	–	–
	0.225	0.1613	94	29.589	29.880	1.061	22.5	39.6	668.4	552.09
	0.225	0.1613	38	29.526	29.733	1.057	28.0	36.0	665.5	549.70
	0.225	0.1613	62	29.571	29.865	1.059	30.0	37.8	643.2	549.18
	0.225	0.1613	161	29.553	29.781	1.058	38.5	36.9	603.1	545.00
CO_2	0.225	0.1613	–	–	28.278	1.041	21.5	21.8	–	–
	0.225	0.1613	–	–	28.254	1.035	21.0	16.5	–	–
	0.225	0.1613	–	–	28.194	1.034	19.0	15.6	–	–
	0.225	0.1613	–	–	28.224	1.039	24.5	20.0	–	–
	0.225	0.1613	20	28.167	28.506	1.050	33.0	29.8.5	585.4	546.10
	0.225	0.1613	75	28.257	28.617	1.054	27.5	33.3	747.2	542.08
	0.225	0.1613	61	28.206	28.770	1.063	43.0	41.3	743.6	549.18
	0.225	0.1613	58	28.242	28.677	1.055	35.5	34.2	759.5	551.04

Table 9: calibration gas piston prober with N_2

$p_{L,N_2}\,(bar)$	$V_{N_2}\,(ml)$
1.018	4.0
1.026	8.0
1.032	14.5
1.038	19.0
1.044	20.5
1.047	24.5
1.056	36.5
1.057	37.0
1.061	40.5
1.063	42.0
1.067	46.0

is based on p_L. As the latter is independent on the atmospheric pressure, the results based on $V_{P,calc,\Delta U}\,(mL)$ are comparable to each other.

1.9 Data calculation

The $p-T$-functions where fitted using RGP (and RKP - functions. In case of constant $T_{counter}$ values of 25° gives

$$\begin{aligned} p_{Ar,25^\circ C} &= (0.008950 * 25)\,bar + 28.84932 bar \\ p_{N_2,25^\circ C} &= \left(29.779175 * 0.999568^{25}\right)\,bar \\ p_{CO_2,25^\circ C} &= \left(28.44393 * 0.999489^{25}\right)\,bar \end{aligned} \tag{3}$$

In case of constant P they are given by

$$\begin{aligned} p_{Ar,25^\circ C} &= (0.048989 * 25)\,bar + 27.902511 bar \\ p_{N_2,25^\circ C} &= (0.053420 * 25)\,bar + 28.306762 bar \\ p_{CO_2,25^\circ C} &= (0.059106 * 25)\,bar + 26.665468 bar \end{aligned} \tag{4}$$

Table 10: p, T and ρ-values from RGP/RKP-functions for Ar, N_2 and CO_2 from experiments at $25°C$ and p, T and ρ-values from p and T starting values;ρ-values are taken from NIST-database[3]

gas	exp. constant $T_{counter}$ $p_{25°C}\,(bar)$	$p_{T_{Start}}\,(bar)$	exp. constant P $p_{25°C}\,(bar)$	$p_{T_{Start}}\,(bar)$
Ar	29.073	29.067	29.127	29.070
N_2	29.460	29.514	29.642	29.493
CO_2	28.082	28.161	28.143	27.951
gas	$T\,(°C)$	$T_{Start}\,(°C)$	$T\,(°C)$	$T_{Start}\,(°C)$
Ar	25.0	23.8	25.0	23.8
N_2	25.0	22.2	25.0	22.2
CO_2	25.0	21.6	25.0	21.9
gas	$\rho\left(\frac{g}{ml}\right)$	$\rho_{Start}\left(\frac{g}{ml}\right)$	$\rho\left(\frac{g}{ml}\right)$	$\rho_{Start}\left(\frac{g}{ml}\right)$
Ar	0.047662	0.047862	0.047752	0.047867
N_2	0.033419	0.03382	0.033626	0.033797
CO_2	0.059112	0.060532	0.059268	0.059869

1.10 Reproducibility of the EFSA-effect

To prove the reproducibility and robustness of the observed effects, a measurement campaign was carried out which implies different individual sample masses for different gases owning a mass difference of ±10 % including the degassing at 150° (see table 11)and measurements of the EFSA-effect at comparable pressures. Every sample used in this study contained a ratio of Carbon to SiO_2 of $1 \pm 0.6\%$. Therefore, differences in the carbon/SiO_2-ratio are within the errors of measurements given above*.

Table 11: Results for different sample masses including degassing at 150 °; the sample masses used for Ar, N_2 and CO_2 are 163.7 and 166.7, 147.5 and 180.8 mg; sample volumes are not included into the calculation; volume sample cell: 15.488ml

gas	$T_{\Delta U}$ ($^\circ C$)	$p_{\Delta U}$ (bar)	$\rho_{\Delta U}$ $\left(\frac{g}{ml}\right)$	$T_{\Delta U=0}$ ($^\circ C$)	$p_{\Delta U=0}$ (bar)	$\rho_{\Delta U=0}$ $\left(\frac{g}{ml}\right)$	$n_{ad,\Delta U}$ ($mmol$)	ΔU (V)	I (mA)
Ar	24.9	29.316	0.048085	25.0	29.850	0.048957	0.338	23.0	24.9
Ar	25.3	29.211	0.047840	24.8	29.622	0.048613	0.300	14.3	24.9
N_2	25.1	29.649	0.033622	24.4	30.066	0.034182	0.3094	29.2	18.7
N_2	24.8	29.607	0.033610	24.5	29.982	0.034074	0.256	16.0	24.0
CO_2	24.6	29.344	0.062520	24.8	29.847	0.063766	0.438	19.0	26.4
CO_2	24.8	29.01	0.061574	24.8	30.045	0.064289	0.478	60.0	18.8

The average values for $n_{ad,\Delta U}$ for Ar, N_2 and CO_2 are 0.319, 0.283 and 0.458 mmol(the second value for CO_2 was halved because a twice as high electric power was applied). The trend follows the values given in table 4 of the main manuscript, where values of 0.225, 0.151 and 0.378 $mmol$ were given. Changes in excess adsorption of Ar, N_2 and CO_2 are herein the result of multiple high temperature degassing processes which is more effective but also leads to enhanced chemisorption and sample detioration (especially in case of CO_2). The trend is also approximately maintained in case of adsorption capacity (1.93, 1.92 and 2.53 $\frac{mmol}{g}$ for Ar, N_2 and CO_2 compared to 1.39, 0.93 and 2.33 $\frac{mmol}{g}$ from the values given in table 3 in the supplementary chapter 1.3.1). Values for Volumes are shown in table 12.

Table 12: Results for different sample masses including degassing at 150 °

gas	sample mass (mg)	$p_{L,\Delta U}$, (bar)	$p_{L,0}$, (bar)	$V_{P,calc,\Delta U}$, (ml)	$V_{P,calc}$, (ml)
Ar	163.7	1.050	1.035	29.8	16.5
Ar	166.7	1.073	1.058	49.8	36.7
N_2	147.5	1.033	1.023	14.7	5.8
N_2	147.5	1.036	1.019	17.4	2.3
CO_2	180.8	1.069	1.025	46.7	7.6
CO_2	180.8	1.060	1.033	38.7	14.6

The average differences in volume are therefore 13.2, 12.0 and 31.6 ml for the gases Ar,

N_2 and CO_2, which reproduces the trend given in table 2 in the manuscript. Deviations and errors herein are a result of the low proportion of ΔV compared to the overall volume (only $\approx 1 - 2\%$) as well as the different volume expansions resulting from different starting pressures and virial coefficients of the gases.

* The investigated system is subject to multiple parameters which influence the numerical results of the measured pressures and volumes. The volume of the sample is much lower compared to the volume of the sample cell, hence a change in pressure can be regarded independent of the sample volume. In general, the sample inside the sample chamber is a point source of heat which is counter-cooled from the outside. Hence, there are temperature gradients in the cell, which are kept while applying the field resulting in a constant sample temperature ($p_{\Delta U}$, $T_{\Delta U}$). Therefore, this condition is only stationary, but not microscopically thermally equilibrated. After switching of the field, the absence of an internal heat source allows a thermal equilibrium ($p_{\Delta U=0}$, $T_{\Delta U=0}$). As the sample determines the heat evolution due to the applied field, the sample mass defines the internal temperature gradients as well as the sample height. The latter herein defines the conductivity, which is higher with increasing height and vice versa. This fact also gives rise to a different field and heat distribution inside the sample. In addition, these factors are also influenced by the gas used, as it owns a specific heat capacity and influence on the conductivity of the sample material due to surface adsorption effects. Therefore, the same effect at different masses would be a proof for the robustness of the results. Furthermore, application of different degassing temperatures or fields can lead to sample deterioration.

2 Simulation of voltage and current flow

Calculations where done using FEMM (Finite Element Method Magnetics) version 4.2. Conductivity of surrounding gas, carbon and SiO_2 was set to 0, 20^5 and 0. Permittivity of surrounding gas, carbon and SiO_2 was set to 1, 23[12] and 3.9. Loss Tangent of Electrical Permittivity of surrounding gas, carbon and SiO_2 was set to 0, 6[13] and 0. Permittivity, con-

ductivity and Loss Tangent of Electrical Permittivity where set equal in x and y direction in every case.

References

(1) Jens Möllmer Adsorption der einen Gase CO2, CH4 und N2 sowie deren Gemische an Mikroporösen Koordinationspolymeren. Dissertation, Universität Leipzig, Leipzig, 21.05.2012.

(2) Gale, W. F.; Totemeier, T. C. *Smithells metals reference book*, 8th ed.; Elsevier/Butterworth-Heinemann: Amsterdam, 2004.

(3) Lemmon, E. W.; Bell, I. H.; Huber, M. L.; McLinden, O. *Thermophysical Properties of Fluid Systems in in NIST Chemistry WebBook, NIST Standard Reference Database Number 69, Eds. P.J. Linstrom and W.G. Mallard*; National Institute of Standards and Technology: Gaithersburg MD, 20899, 2024.

(4) Siebel, K. Über die Änderung des elektrischen Widerstandes von Kohle durch Gasabsorption. *Zeitschrift fr Physik* **1921**, *4*, 288–299.

(5) Barroso Bogeat, A. Understanding and Tuning the Electrical Conductivity of Activated Carbon: A State-of-the-Art Review. *Critical Reviews in Solid State and Materials Sciences* **2019**, 1–37.

(6) Wang, Y.; Alsmeyer, D. C.; McCreery, R. L. Raman spectroscopy of carbon materials: structural basis of observed spectra. *Chemistry of materials : a publication of the American Chemical Society* **1990**, *2*, 557–563.

(7) Tan, P.; Hu, C.; Dong, J.; Shen, W.; Zhang, B. Polarization properties, high-order Raman spectra, and frequency asymmetry between Stokes and anti-Stokes scattering of Raman modes in a graphite whisker. *Physical Review B* **2001**, *64*, 959.

(8) Escribano, R.; Sloan, J. J.; Siddique, N.; Sze, N.; Dudev, T. Raman spectroscopy of carbon-containing particles. *Vibrational Spectroscopy* **2001**, *26*, 179–186.

(9) Kawashima, Y.; Katagiri, G. Fundamentals, overtones, and combinations in the Raman spectrum of graphite. *Physical review. B, Condensed matter* **1995**, *52*, 10053–10059.

(10) Fantini, C.; Jorio, A.; Souza, M.; Saito, R.; Samsonidze, G. G.; Dresselhaus, M. S.; Pimenta, M. A. Steplike dispersion of the intermediate-frequency Raman modes in semiconducting and metallic carbon nanotubes. *Carbon* **2005**, *72*, 027401.

(11) Marinescu, M. *Elektrische und magnetische Felder*; Springer Berlin Heidelberg: Berlin, Heidelberg, 2012.

(12) Atwater, J. E.; Wheeler, R. R. Complex permittivities and dielectric relaxation of granular activated carbons at microwave frequencies between 0.2 and 26 GHz. *Carbon* **2003**, *41*, 1801–1807.

(13) Yu, Z. Research on absorbing performance of activated carbon. *IOP Conference Series: Materials Science and Engineering* **2019**, *563*, 022023.
